# Improving the Biological Properties of Thrombin-Binding Aptamer by Incorporation of 8-Bromo-2′-Deoxyguanosine and 2′-Substituted RNA Analogues

**DOI:** 10.3390/ijms242115529

**Published:** 2023-10-24

**Authors:** Antonella Virgilio, Daniela Benigno, Carla Aliberti, Valentina Vellecco, Mariarosaria Bucci, Veronica Esposito, Aldo Galeone

**Affiliations:** Department of Pharmacy, University of Naples Federico II, 80131 Napoli, Italy; antonella.virgilio@unina.it (A.V.); daniela.benigno@unina.it (D.B.); vellecco@unina.it (V.V.); mrbucci@unina.it (M.B.); galeone@unina.it (A.G.)

**Keywords:** G-quadruplex, thrombin binding aptamer, 8-bromo-2′-deoxyguanosine, RNA analogues, anticoagulant activity

## Abstract

Thrombin-binding aptamer (TBA) is one of the best-known G-quadruplex (G4)-forming aptamers. By adopting its peculiar chair-like G4 structure, TBA can efficiently bind to thrombin, thus producing an anticoagulant effect. The major limit to its therapeutic application is represented by its poor thermal and biological resistance. Therefore, numerous research studies have been focused on the design of TBA analogues with chemical modifications to improve its pharmacokinetic and pharmacodynamic properties. To maintain the functional recognition to protein surface on which TBA anticoagulant activity depends, it is essential to preserve the canonical antiparallel topology of the TBA quadruplex core. In this paper, we have designed three TBA variants with modified G-tetrads to evaluate the effects of nucleobase and sugar moiety chemical modifications on biological properties of TBA, preserving its chair-like G-quadruplex structure. All derivatives contain 8-bromo-2′-deoxyguanosine (G^Br^) in syn positions, while in the anti-positions, locked nucleic acid guanosine (G^LNA^) in the analogue TBABL, 2’-*O*-methylguanosine (G^OMe^) in TBABM, and 2’-F-riboguanosine (G^F^) in TBABF is present. CD (Circular Dichroism), CD melting, 1H-NMR (Nuclear Magnetic Resonance), and non-denaturing PAGE (Polyacrylamide Gel Electrophoresis), nuclease stability, prothrombin time (PT) and fibrinogen-clotting assays have been performed to investigate the structural and biological properties of these TBA analogues. The most interesting results have been obtained with TBABF, which revealed extraordinary thermal stability (T_m_ approximately 40 °C higher than that of TBA), anticoagulant activity almost doubled compared to the original aptamer, and, above all, a never-observed resistance to nucleases, as 50% of its G4 species was still present in 50% FBS at 24 h. These data indicate TBABF as one of the best TBA analogue ever designed and investigated, to the best of our knowledge, overcoming the main limitations to therapeutic applications of this aptamer.

## 1. Introduction

**Figure 1:**
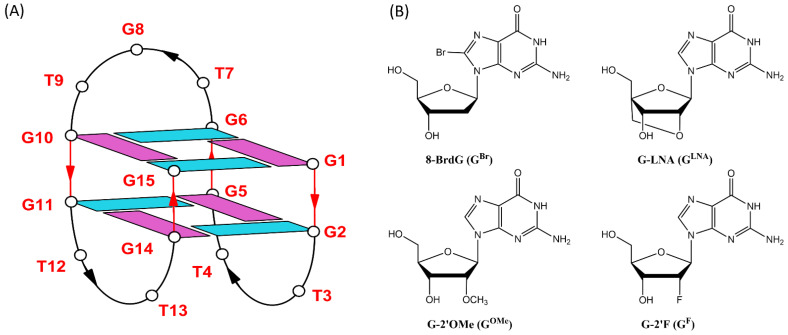
Schematic representation of the TBA G-quadruplex. Deoxyguanosines in *syn*- and *anti-glycosidic* conformations are in purple and light blue, respectively (**A**); chemical structures of 8-bromo-2′-deoxyguanosine (G^Br^), locked nucleic acid guanosine (G^LNA^), 2’-*O*-methylguanosine (G^OMe^) and 2’-F-riboguanosine (G^F^) (**B**).

Thrombin-binding aptamer (TBA) is a 15-mer oligodeoxynucleotide (5′-GGTTGGTGTGGTTGG-3′) that has stood out from the beginning due to its remarkable anticoagulant properties [1,2] and, more recently, its antiproliferative potential [3,4], as it is the subject of several therapeutic research studies [5]. Furthermore, considering that the COVID-19 pandemic emergency brought out the need to use low-dose anticoagulants in COVID-19-hospitalized patients to prevent thrombosis, the interest in non-toxic but biologically stable and economically viable alternatives to traditional anticoagulants, such as heparin, was recently felt again, and even more so [6]. According to X-ray and NMR spectroscopy studies, TBA folds into a chair-shaped, monomolecular, antiparallel G-quadruplex structure, consisting of two stacked G-tetrads linked by three lateral loops (two TT loops and one TGT loop) (Figure 1A) [7,8]. By adopting this peculiar chair-like G4 structure, TBA can efficiently bind the thrombin exosite I, acting as a protease activity modulator, thus inhibiting fibrin clot formation [7,9,10,11], with an EC50 value of 20 nM in a purified fibrinogen-clotting assay [1]. Unfortunately, the therapeutical development of the unmodified TBA as an anticoagulant agent halted, mainly due to some critical aspects, like the rather poor stability from both a thermodynamic and a biological point of view, so clinical trials evaluating TBA as anticoagulant for cardiovascular settings, such as coronary artery bypass graft (CABG) surgery, were halted after Phase I studies due to suboptimal dosing profiles [12,13,14]. Taking in account the inexpensive and efficient chemical synthesis, small size, and the lack of side effects, and reversibility of action pointed out by preclinical and clinical studies involving TBA and other anti-coagulant aptamers [15,16,17], a significant part of the research focused on the development of suitable analogues of TBA with chemical modifications aimed at increasing thermal and biological resistance since the natural counterpart is mostly degraded in 1 h in 10% FBS [18]. Furthermore, starting from the TBA’s ability to fold unequivocally into an active well-known antiparallel conformation because of its short oligonucleotide sequence [19], post-SELEX modifications represent an effective strategy to improve both the interaction with the target protein based on the knowledge of the structure-activity relationships (SARs) [20,21,22] and its poor pharmacokinetic properties in vivo, finally leading to potentially more promising therapeutic agents.

Numerous synthetic modifications have been performed to improve the pharmacological properties of TBA. Suitable chemical modifications can involve guanine tetrads, loop nucleotides, and sugar and phosphodiester linkages [23,24,25,26]. Most of these modified aptamers revealed enhanced thermal stability but similar or decreased anticoagulant activities in comparison to the native one [5,27].

It is now accepted that the G4 aptamer core, formed by stacked G-tetrads, is principally responsible of their stability, while the loop residues play a major role in the target protein recognition. Consequently, in designing modified aptamers to modulate their biological properties without affecting their stability, in most cases, modifications are focused on loop residues. However, in a recent paper, Svetlova et al. studied the effect of modifications in the TBA quadruplex core on the ability of this aptamer to interact with thrombin, suggesting that the canonical antiparallel topology of the two G-quartets is required to maintain the functional recognition of the protein surface [28].

Generally, TBA modifications can be grouped into two categories: (1) sugar/phosphate backbone modifications, such as locked nucleic acid (LNA) [29,30], 2′-fluoro-arabinonucleic acid (2′-F-ANA) [18] or unlocked nucleic acid (UNA) [31,32]; and (2) nucleobase modifications, such as alkylation, phenylation or bromination [28,33,34,35]. These strategies provided the most interesting results when the chemical modifications contributed to the stabilization of specific glycosidic bond conformations in the TBA G-tetrads.

In the present study, we have prepared three TBA variants with modified tetrads to evaluate the synergic effects of nucleobase and sugar-moiety chemical modifications on the biological properties of TBA, preserving its antiparallel chair-like G-quadruplex structure. In all variants, we have substituted the G-core guanosines that are in syn positions with 8-bromo-2′-deoxyguanosine (G^Br^), while the guanosines in the anti-positions have been replaced with locked nucleic acid guanosine (G^LNA^) in TBABL, 2’-*O*-methylguanosine (G^OMe^) in TBABM, and 2’-F-riboguanosine (G^F^) in TBABF (Figure 1B). G^Br^ is a nucleotide known to stabilize a syn conformation of the glycosidic bond by steric hindrance between the bromine and the deoxyribose moiety. Furthermore, it has been demonstrated that the introduction of this modified guanosine in appropriate positions increases the affinity of TBA [28,35]. LNA is a modified RNA nucleotide in which the ribose moiety contains an extra bridge connecting the 2’ oxygen and 4’ carbon. The bicyclic structure of LNA forces the sugar to adopt the C3′-endo conformation, and nucleotides with this conformation prefer to adopt the anti-glycosidic bond configuration [36]. Also 2’-*O*-methyl nucleotides (OMe) with their C3’-endo sugar pucker conformation and anti-glycosidic angle were previously used to used to selectively substitute the guanine residues of G-tetrads of TBA, revealing that single substitutions for anti-dG residues could preserve the G-quadruplex in a K^+^ environment [37]. The 2′-deoxy-2′-fluoro-riboguanosine (GF) represents another useful chemical tool for manipulating G-quadruplex folding by anti-position-favoring substitutions [38], as well as increasing the resistance of modified oligonucleotides to degradation by nucleases [39], as is the case with most 2’-modified RNA aptamers.

Therefore, assuming that selectively placed G^Br^ and G^LNA^, G^OMe^ or G^F^ would affect the G-quadruplex folding topology of TBA, which could potentially influence its activity, CD, CD melting, 1H-NMR, non-denaturing PAGE, nuclease stability, prothrombin time (PT) and fibrinogen-clotting assays were performed to investigate the structure, thermodynamic and biological stability, and the anticoagulant activity of the modified TBA sequences reported in Table 1.

## 2. Results

### 2.1. Structural Insights of Investigated TBA Derivatives

**Figure 2:**
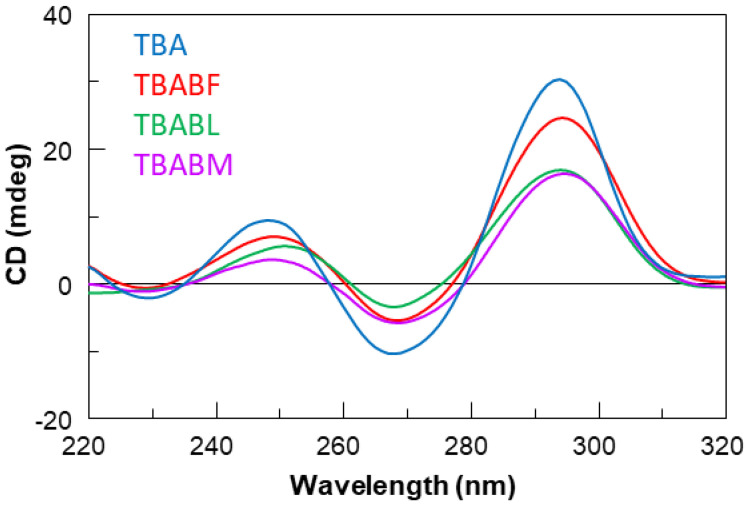
CD spectra at 20 °C of TBA and its investigated analogues.

**Figure 3:**
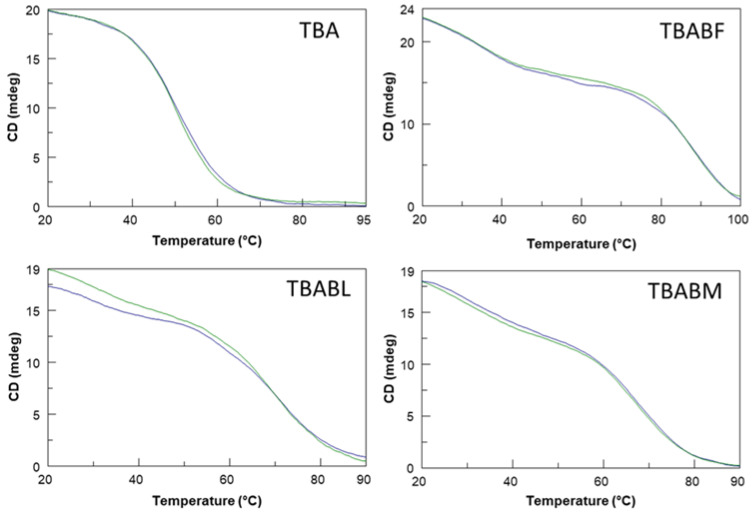
CD melting (blue) and annealing (green) profiles of the G-quadruplex formed by TBA, TBABF, TBABL and TBABM. See Materials and Methods for experimental details.

**Figure 4:**
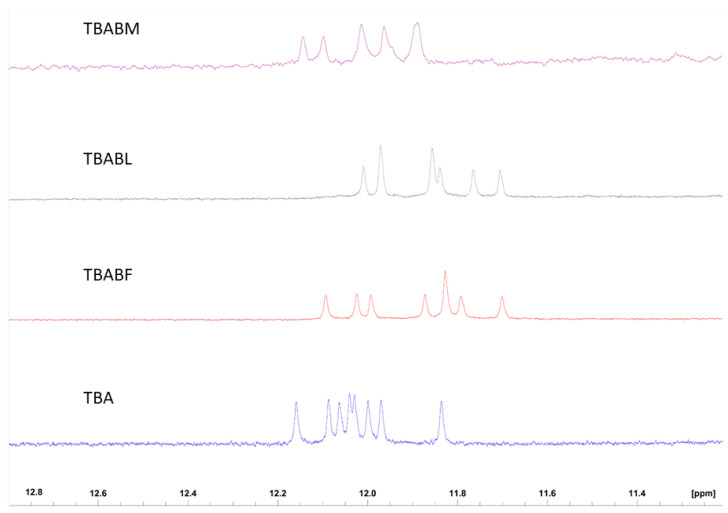
Imino proton regions of the ^1^H-NMR spectra (700 MHz) of TBA and its investigated analogues.

The three modified TBA sequences were investigated by circular dichroism in order to test their ability to adopt a G-quadruplex conformation similar to that of the parent one in the K^+^-containing buffer utilized [40]. It is well known that TBA folds in a monomolecular antiparallel “chair-like” G-quadruplex structure characterized by two stacked *syn-anti-*G-tetrads and three lateral loops. All the ODN analogues showed CD profiles nearly comparable to each other, apart from slight differences in intensity, and closely to that of TBA, which is characterized by two positive bands around 247 and 294 nm, and a negative one around 266 nm. These data clearly suggest that the modifications do not prevent TBA derivatives from adopting the antiparallel ‘chair-like’ G-quadruplex structure typical of the unmodified aptamer (Figure 2). CD melting measurements were also used to evaluate the thermal stability of the modified ODNs (Figure 3). The mostly sigmoidal CD heating profiles of TBA derivatives allowed us to confidently measure the melting temperatures (Tm) (Table 1), which are all much higher than that of TBA (50 °C) [25]. Interestingly, the melting curve analysis shows that TBABF is characterized by a melting temperature of 89 °C, thus clearly indicating an outstanding thermal stability for this structure. Furthermore, the melting and annealing profiles of each TBA derivative are almost superimposable (Figure 3), as is the case of the unmodified aptamer. The absence of hysteresis between the heating and cooling profiles in all cases, thus indicating the fast equilibrium kinetics of the system in the experimental conditions, would suggest the occurrence of monomolecular G-quadruplex structures. To confirm this, we performed a non-denaturing PAGE analysis (Figure S1). The electrophoretic profile clearly indicated that all the TBA analogues form G-quadruplex structures comparable to that of the original one since they showed migrating bands only slightly slower than the canonical TBA due to their higher molecular weight than the unmodified aptamer. Furthermore, the presence in each lane of a single bandwidth electrophoretic motility almost similar to that of TBA strongly suggested that all investigated analogs are able to adopt a single well-defined G-quadruplex conformation with the same molecularity of the parent one, which is in agreement with the CD data. The ability of all the reported TBA derivatives to fold into TBA-like antiparallel quadruplexes was also assessed by NMR spectroscopy. The NMR samples were prepared at a concentration of about 1.0 mM in strands (0.6 mL, 90% H_2_O/10% D_2_O), with a 10 mM potassium phosphate, 70 mM KCl and 0.2 mM EDTA (pH 7.0) buffer. The samples were annealed by heating them for 5–10 min at 80 °C and allowing them to slowly cool down (10–12 h) to room temperature. The solutions were equilibrated for at least 1 week at 4 °C, and then their ^1^H-NMR spectra were recorded at 25 °C. A completed annealing process was assured by the achievement of super-imposable ^1^H-NMR spectra on changing time. ^1^H-NMR spectra (Figure 4) indicate that, in the conditions used here, the modified oligomers mainly form a single well-defined hydrogen-bonded conformation, showing a related signal distribution for all the variants in the region of 11.6–12.2 p.p.m., which is attributable to eight exchangeable protected imino protons involved in the formation of two G-tetrads. These data suggest that all the new derivatives fold in a conformation very similar to the parent TBA, thus clearly hinting that regardless of the modification type, the variations are not able to significantly affect the topology of the original structure, with some slight differences that cannot be investigated because of the partial signal overlapping and the lack of the H8 of Gs in the syn position of TBA, as it is replaced by a bromine atom.

### 2.2. Nuclease Stability Assay

**Figure 5:**
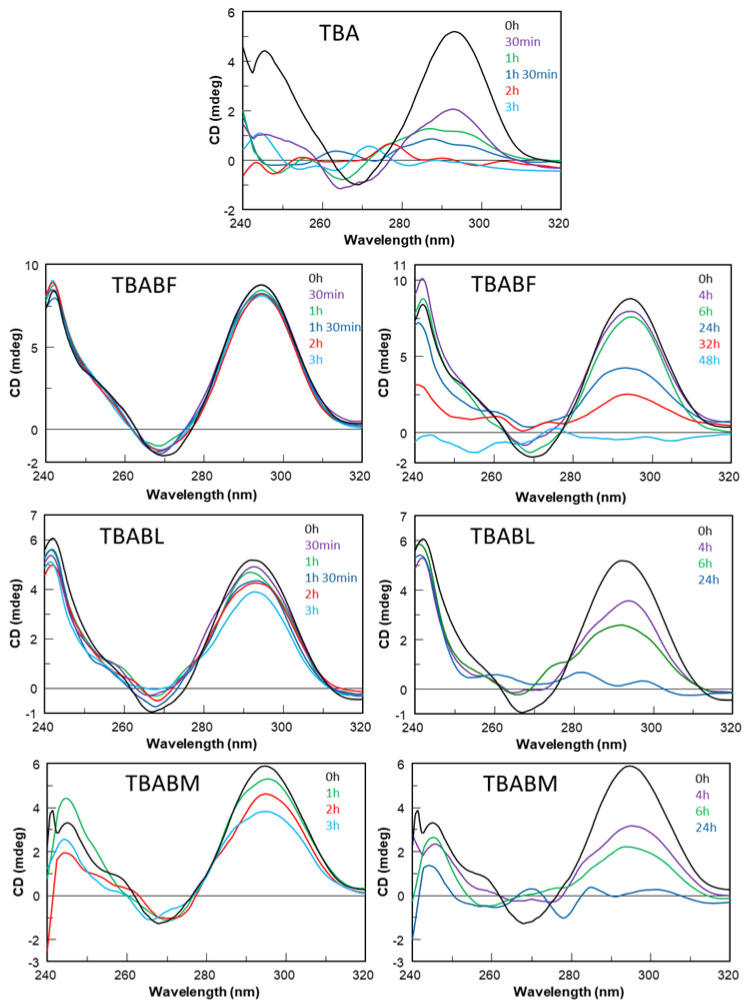
CD spectra of investigated ODNs in 50% Fetal Bovine Serum (FBS) diluted with Dulbecco’s Modified Eagle’s Medium (DMEM) and registered at different times at 37 °C. See the main text and the Section 4 for details.

To exam the resistance in biological environments, all the analogues have been tested in comparison to the natural counterpart through a degradation assay in Fetal Bovine Serum (FBS) and analyzed by circular dichroism recorded at 37 °C in 50% FBS at different times (range 0–48 h) for each sample until the disappearance of the CD signal attributable to the G-quadruplex. In Figure 5, on the left, CD spectra of the modified aptamers registered in the same range time of natural TBA are reported. Results clearly indicated that in these conditions, the three modified aptamers are rather resistant to nucleases for up to 3 h, while their natural counterpart is completely degraded in 2 h. The most interesting results were obtained for TBABF, which revealed more than 90% of undegraded species at 3 h, thus indicating a noteworthy improvement in nuclease resistance compared to both TBA and other analogues. Under the same experimental conditions, all modified analogues CD spectra were also acquired in the 4–24 h time range (Figure 5 on the right). These data confirmed the TBABF’s extraordinary resistance as 50% and 30% of its G4 species is still present at 24 h and 32 h, respectively, while TBABM and TBABL are completely degraded at 24 h, as indicated by the absence of G-quadruplex CD signals in the 240–320 nm region after subtraction of the background scan (50% FBS in DMEM). These data clearly indicate that the modified aptamer TBABF is endowed with a remarkable stability in biological environments.

### 2.3. Anticoagulant Activity

**Figure 6:**
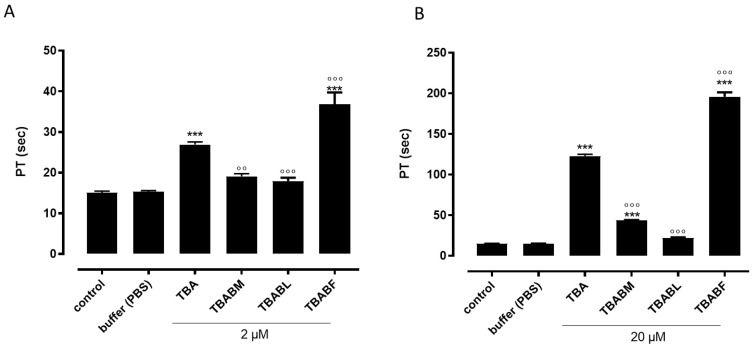
Concentration-dependent response, following 15 min incubation of TBA and its investigated analogues with human plasma at 2 (**A**) or 20 μM (**B**). PT values are expressed in seconds. Each measurement was performed in triplicate and shown as the mean ± SEM. *** = *p* < 0.001 vs. vehicle, °° = *p* < 0.01, °°° = *p* < 0.001 vs. TBA.

**Figure 7:**
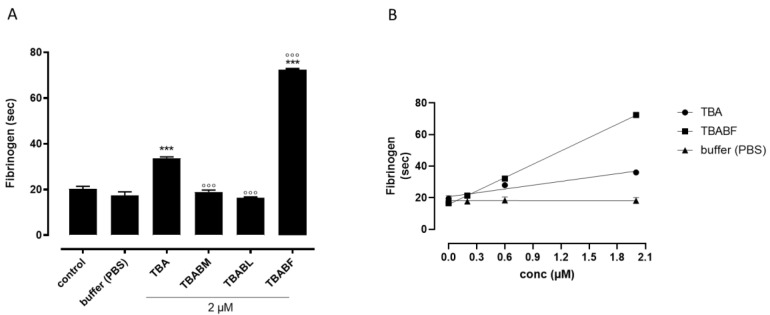
Fibrinogen-clotting time of TBA and its investigated analogues on human plasma. (**A**) Fibrinogen-clotting time of human plasma following 15 min incubation of TBA and its investigated analogues at 2 µM. (**B**) Concentration-dependent response of TBA and TBABF on fibrinogen-clotting time. Time value was expressed in seconds. Each measurement was performed in triplicate and shown as the mean ± SEM *** = *p* < 0.001 vs. vehicle, °°° = *p* < 0.001 vs. TBA.

To evaluate the possible anticoagulant properties of the TBA analogues, ODNs were subjected to PT assay, and their activity was compared to that of TBA (Figure 6). The results clearly showed that among all the modified aptamers, TBABL was completely devoid of anticoagulant activity at both the concentrations used (2–20 µM) (Figure 6A,B). Conversely, TBABM preserved its anticoagulant activity to some extent. In detail, the TBABM analogue, at the lowest concentration used (2 µM), did not show any anticoagulant property (Figure 6A); however, when the same compound was tested at the highest concentration (20 µM), the TBABM displayed an anticoagulant profile, even though it was to a lesser extent compared to the original TBA (Figure 6B). Finally, the evaluation of PT in plasma incubated with TBABF revealed an increased PT value that was significantly higher when compared to its natural counterpart TBA at both the concentrations used (Figure 6A,B). To validate the results obtained from the PT assay, we also performed the fibrinogen-clotting assay (Fibrinogen Clauss) on human plasma. As shown in Figure 7A, among the tested ODNs, TBABF was as the best anticoagulant. The fibrinogen-clotting values measured in the presence of TBABM and TBABL confirmed that these modified ODNs were completely unable to inhibit thrombin; therefore, the fibrinogen values pointed to a trend of the antithrombin activities that was almost in agreement with the anticoagulant activities measured in the PT assay. To compare the inhibitory activity of TBABF with that of TBA, we a concentration-response curve for each of them. When fibrinogen was added to the plasma incubated with TBABF or original TBA, the clotting time was prolonged in a concentration-dependent manner (0.2–0.6 and 2 µM) compared to the vehicle. Notably, TBABF showed a substantial increase in its ability to inhibit thrombin activity, whereas the inhibitory activity of TBA was only slightly increased (Figure 7B), confirming that TBABF is the best inhibitor. Specifically, the TBABF-induced fibrinogen-clotting time was more than doubled compared to the unmodified TBA at the highest concentration used (2 µM) (72.4 ± 0.43 s vs. 33.8 ± 0.51 s, TBABF vs. TBA, respectively), suggesting that the TBABF has a higher affinity to bind to thrombin than the original TBA. These results are comparable to those obtained by PT assay, implying a very interesting thrombin-binding ability in TBABF analogue.

## 3. Discussion

Among aptamers adopting a G-quadruplex structure, TBA is one of the most investigated, and it is still the subject of several studies. A compelling number of investigations has proven that the chemico-physical and biological properties of TBA can be regulated by site-specific replacements of appropriate residues of the loops and/or of the central G-core. Depending on the type of post-SELEX modifications used and the position in the sequence, varying results have been obtained. However, most of the loop modifications, by altering the conformation of the aptamer, which has been optimized by the SELEX process to bind thrombin, can revert the favorable properties, except for T7 residue, which was identified as an efficient modulator of the quadruplex thermodynamic stability, binding affinity, and biological properties of TBA, as well as UNA monomers [31,32]. Since the thermal stability of a G-quadruplex structure and the ability of this aptamer to interact with thrombin depend on the core of stacked G-tetrads to some extent, an interesting approach to obtain more stable and active TBA derivatives is the modification of the GQ core retaining thechair-like topology.

Therefore, in this paper, we have investigated the structural properties and the biological activities of three TBA analogues in which the G-core guanosines in syn positions have been replaced with 8-bromo-2′-deoxyguanosine (G^Br^), while the guanosines in the anti ones were replaced with locked nucleic acid guanosine (G^LNA^) in TBABL, 2’-*O*-methylguanosine (G^OMe^) in TBABM, and 2’-F-riboguanosine (G^F^) in TBABF (Table 1). These TBA derivatives characterized by modified tetrads have been designed with the aim of exploiting the synergic effects of nucleobase and sugar-moiety chemical modifications on the biological properties of TBA, preserving the antiparallel chair-like G-quadruplex structure. In order to confirm this issue, CD profiles, CD melting and annealing, 1H-NMR spectra, non-denaturing PAGE analysis were acquired. One of the most straightforward applications of CD to G-quadruplex investigation is to determine whether a sequence analogue of a parent ODN is similarly folded, or if a chemical modification can affect its conformation or stability. The CD profiles of the TBA derivatives are shown together in Figure 2 in comparison with that of the unmodified aptamer, all exhibiting the typical profile of an antiparallel G-quadruplex in which anti and syn guanosines alternate along the strands, being characterized by two positive bands at 247 and 294 nm, and a negative one at 266 nm. The CD heating/cooling curves can provide additional information about the G-quadruplex structure adopted by the modified ODNs and, in particular, to determine the effects of chemical modifications on G-quadruplex thermal stability. A comparison of the estimated melting temperatures (Tm) listed in Table 1 shows that all modified analogues adopt G-quadruplex structures that are much more stable than that of the unmodified parent aptamer, noticeably indicating the extraordinary thermal stability of TBABF (89 °C). Importantly, similarly to the parent TBA and other TBA derivatives [41], CD melting/annealing profiles of the three modified aptamers are almost superimposable (Figure 3), revealing fast equilibrium kinetics of the system in the experimental conditions used, which suggest the presence of monomolecular G-quadruplex structures for these modified aptamers as well. In order to further verify this point, the TBA derivatives were investigated further by non-denaturing PAGE and comparing to their natural counterpart. The electrophoretic data (Figure S1) clearly show that all the TBA analogues form G-quadruplex structures with electrophoretic motilities very similar to those of the unmodified aptamer. The presence of single bands with slightly slower migration profiles, attributable to the presence of bromines and 2′substituents, strongly suggests the occurrence of G-quadruplex conformations comparable to that of the original TBA and with the same molecularity for all three derivatives, which is in agreement with the CD data. Moreover, the close similarity of the 1H NMR profiles of the modified ODNs and their unmodified version confirmed that TBA analogues adopt a TBA-like antiparallel G-quadruplex conformation, showing almost superimposable imino proton regions, confirming the presence of G-quadruplex structures (11.6–12.2 ppm) characterized by two G-tetrads. The resistance in biological environments of nucleic acid aptamers is one of the most critical requirements for their potential use in therapeutic applications. An aptamer with effective stability under physiological conditions can be promptly applied for biomedical treatments without further optimization with a significant reduction in expenses. The introduction of modified residues into an aptamer can influence their biostability. Consequently, to test the susceptibility on nucleases’ digestion, all three TBA derivatives were undergone to a degradation assay in Fetal Bovine Serum (50% FBS) and analyzed at different times by CD, in comparison with the unmodified aptamer (Figure 5). The TBA analogues persist as undegraded G4 structures for the most part up to 3 h, while the unmodified aptamer degrades totally in 2 h in serum, showing that G-core substitutions affect the aptamers’ nuclease resistance. Noteworthily, the modified aptamer TBABF shows an outstanding stability in biological environments, as about 50% and 30% of its G4 species is still present at 24 h and 32 h, suggesting that 2’-F-riboguanosine (G^F^) substitutions of the anti-guanosines of G-tetrads significantly influence the aptamers’ susceptibility on nucleases digestion, which is probably related to its remarkable thermal stability. The results from PT and fibrinogen-clotting assays (Figure 6 and Figure 7) allowed us to determine that TBABL and TBABM anticoagulant activities have turned out lower than the natural TBA, thus being completely unable to inhibit thrombin. Differently, TBABF has shown a significant enhancement in anticoagulant activity, which has never been observed before, almost doubling at high concentrations compared to the original aptamer, indicating TBABF as the best inhibitor with a higher affinity to bind thrombin and a much more pronounced nuclease resistance than the parent TBA.

## 4. Materials and Methods

### 4.1. Oligonucleotide Synthesis and Purification

The ODNs listed in Table 1 were synthesized by an ABI 394 DNA synthesizer using solid-phase β-cyanoethyl phosphoramidite chemistry at the 10 µmol scale. The synthesis was carried out using normal 3′-phosphoramidites (Link Technologies, Glasgow, UK). The modified monomers were introduced in the sequences using commercially available 5’-dimethoxytrityl-N2-dimethylaminomethylidene-8-bromo-2’-deoxyGuanosine,3’-[(2-cyanoethyl)-(*N*,*N*-diisopropyl)]-phosphoramidite; 5’-dimethoxytrityl-*N*-dimethylformamidine-(2’-*O*, 4’-C methylene)-Guanosine,3’-[(2-cyanoethyl)-(*N*,*N*-diisopropyl)]-phosphoramidite; 5’-dimethoxytrityl-*N*-isobutyryl-Guanosine,2’-*O*-methyl,3’-[(2-cyanoethyl)-(*N*,*N*-diisopropyl)]-phosphoramidite; and 5’-dimethoxytrityl-*N*-isobutyryl-deoxyGuanosine,2’-fluoro-3’-[(2-cyanoethyl)-(*N*,*N*-diisopropyl)]-phosphoramidite (Glen Research, Sterling, VA, USA). For all ODNs, a universal support was used. The oligomers were detached from the support and deprotected by treatment with concentrated aqueous ammonia at room temperature for 24. The combined filtrates and washings were concentrated under reduced pressure, redissolved in H_2_O, analyzed, and purified by high-performance liquid chromatography on a Nucleogel SAX column (Macherey-Nagel, Duren, Germany, 1000-8/46) using buffer A (20 mM NaH_2_PO_4_/Na_2_HPO_4_ aqueous solution (pH 7.0) containing 20% (*v*/*v*) CH_3_CN) and buffer B (1 M NaCl and 20 mM NaH_2_PO_4_/Na_2_HPO_4_ aqueous solution (pH 7.0) containing 20% (*v*/*v*) CH_3_CN). A linear gradient from 0% to 100% B for 45 min and a flow rate of 1 mL/min were used. The fractions of the oligomers were collected and successively desalted using Sep-pak cartridges (C-18). The isolated oligomers proved to be >98% pure by NMR.

### 4.2. CD Spectroscopy

CD samples of the oligonucleotides reported in Table 1 were prepared at an ODN concentration of 50 µM using a potassium phosphate buffer (10 mM KH_2_PO_4_/K_2_HPO_4_ and 70 mM KCl, pH 7.0) and submitted to the annealing procedure (heating at 90 °C and slowly cooling at room temperature). The CD spectra of all quadruplexes and CD melting curves were registered on a Jasco 715 CD spectrophotometer (Jasco, Tokyo, Japan). For the CD spectra, the wavelength was varied from 220 to 320 nm at a 100 nm min^−1^ scan rate, and the spectra recorded a response of 4 s at 1.0 nm bandwidth and normalized by the subtraction of the background scan with buffer. The temperature was kept constant at 20 °C with a thermoelectrically controlled cell holder (Jasco PTC-348). CD melting and annealing curves were registered as a function of temperature (range: 20 °C–90 °C) for all G-quadruplexes, annealed as previously reported, at their maximum Cotton effect wavelengths. The CD data were recorded in a 0.1 cm pathlength cuvette with a scan rate of 30 °C/h.

### 4.3. Gel Electrophoresis

All oligonucleotides were analyzed by non-denaturing PAGE. All oligonucleotide samples were prepared at an ODN concentration of 50 µM by using a potassium phosphate buffer (10 mM KH_2_PO_4_/K_2_HPO_4_, 70 mM KCl, pH 7.0) and submitted to the annealing procedure (heating at 90 °C and slowly cooling at room temperature). Each oligonucleotide was loaded onto a 20% polyacrylamide gel containing Tris–Borate-EDTA (TBE) 2.5× and 20 mM KCl. The run buffer was TBE 1× containing 50 mM KCl. For all samples, a solution of glycerol/TBE 10× was added just before loading. Electrophoresis was performed at 8 V/cm at a temperature close to 10 °C. Bands were visualized by UV shadowing.

### 4.4. NMR Spectroscopy

NMR samples were prepared at a concentration of approximately 1 mM in 0.6 mL (H_2_O/D_2_O 9:1 *v*/*v*) of buffer solution with 10 mM KH_2_PO_4_/K_2_HPO_4_, 70 mM KCl and 0.2 mM EDTA (pH 7.0). All the samples were heated for 5–10 min at 90 °C and slowly cooled (10–12 h) to room temperature. The solutions were equilibrated for several hours at 4 °C. The annealing process was assumed to be complete when the ^1^H NMR spectra were superimposable on changing time. NMR spectra were recorded at 25 °C by employing a 700 MHz Bruker spectrometer (Bruker-Biospin, Billerica, MA, USA). Proton chemical shifts were referenced to the residual water signal, resonating at 4.78 ppm (25 °C, pH 7.0). Water suppression was achieved using the excitation sculpting with the gradient routine included in the “zgesgp” pulse sequence [42]. NMR data processing was performed by using the vendor software TOPSPIN 4.1.4 (Bruker Biospin Gmbh, Rheinstetten, Germany).

### 4.5. Nuclease Stability Assay

Nuclease stability assay of all ODNs was conducted in 50% Fetal Bovine Serum (FBS) diluted with Dulbecco’s Modified Eagle’s Medium (DMEM) at 37 °C and studied by CD analysis. An approximately 14 nmol of stock solution of each ODN (~2 O.D.U.) was evaporated to dryness under reduced pressure and then incubated with 500 μL 50% FBS at 37 °C. The degradation patterns were analyzed by monitoring the CD signal decrease in each sample at 37 °C, as a function of time. CD spectra at different times for each sample were recorded at 37 °C using a Jasco 715 spectrophotometer equipped with a Peltier temperature control system (Jasco, Tokyo, Japan). Data were collected from 240 to 320 nm with a 1 s response time and a 1 nm bandwidth using a 0.1 cm quartz cuvette. Each spectrum shown is corrected for the spectrum of the reaction medium (50% FBS in DMEM).

### 4.6. Prothrombin (PT) Time

The PT assay was performed on human plasma with the Start Max analyzer (Stago) by using a specific kit, namely neoplastine Cl plus (Stago, Asnieres sur Seine, France). The reagent was precalibrated. Two levels of human control (STA Coag Control N and P) were used for daily quality control assessments and analytical performance evaluations. Reagents and controls were reconstituted according to the manufacturer’s instructions. Briefly, this method relies on the high sensitivity of thromboplastin reagent based on recombinant human tissue factors. The addition of neoplastine to the plasma, in the presence of calcium ions, initiates the activation of the extrinsic pathway that culminates with the conversion of fibrinogen into fibrin and, in turn, with the formation of a solid gel. In our experimental conditions, each ODN or vehicle was incubated with 50 μL of plasma at 37 °C for 15 min, and then 100 μL of the kit solution containing neoplastine was added, with the consequent activation of the extrinsic pathway. In detail, for the evaluation of PT at the concentration of 20 μM, 1 μL of the ODN solution (1 mM) or vehicle (phosphate-buffered saline (PBS)) was added to the microtube. For the evaluation of PT at 2 μM, 1 μL of a diluted solution (0.1 mM ODN solution in PBS buffer) was added to the microtube. The PT measurement was produced in triplicate, and the average and the standard error values were calculated and expressed in seconds. The basal clotting time was evaluated by measuring the clotting time in the presence of vehicle.

### 4.7. Fibrinogen-Clotting Assay

The fibrinogen-clotting time (Fibrinogen Clauss) was measured by using a STARTMAX System with a specific kit, namely liquid Fib (STAGO, Asnieres sur Seine, France). The procedure was performed according to the manufacturer’s instructions. To measure the clotting time in the absence of any inhibitor (i.e., the basal clotting time), fibrinogen liquid solution was added to the plasma that was previously incubated at 37 °C for few minutes. In our experimental conditions, each ODN or vehicle was incubated with 150 μL of plasma at 37 °C for 15 min and then 50 μL of the kit solution containing fibrinogen was added, with the consequent formation of the fibrin clot. In detail, for the evaluation of PT at a concentration of 2 μM, 3 μL of the ODN solution (0.1 mM) or vehicle (phosphate-buffered saline (PBS)) was added to the microtube. The fibrinogen-clotting measurement was produced in triplicate, and the average and the standard error values were calculated and expressed in seconds.

## 5. Conclusions

In brief, we have investigated structural and biological properties of three TBA analogues, in which G^Br^ and G^LNA^, G^OMe^, or G^F^ have been selectively placed in syn- and anti-positions of the TBA G-quadruplex central core. Within the broad panorama of the straightforward changes on this aptamer, in this study, the synergic effect of different chemical modifications has been evaluated with the aim of obtaining new anti-thrombin aptamers able to overcome the biggest limit to the therapeutic application of TBA, namely the poor resistance in biological environment. As modified residues are able to replace the canonical Gs, all commercially available monomers have been chosen, in order to achieve aptamers that are easy to be produced.

The obtained data revealed that all derivatives preserved the antiparallel chair-like G-quadruplex structure of the unmodified analogue and all the chemical modifications contributed favorably to the thermal stability of these G-quadruplexes. However, the most interesting results have been obtained with TBABF. Indeed, this analogue revealed an extraordinary thermal stability, showing a Tm approximately 40 °C higher than that of TBA, an anticoagulant activity at high concentrations that is almost doubled compared to the original aptamer and, above all, a never-observed resistance to nucleases, as about 50% of its G4 species was still present in 50% FBS at 24 h.

These data indicate TBABF as the best TBA analogue to have been designed, to the best of our knowledge, overcoming the main limitation to the therapeutic application of this aptamer. Other G-quadruplex forming aptamers will be subjected to the same specific chemical modification strategy, i.e., the specific and simultaneous substitution of G-*syn* and G-*anti* with appropriate monomers that are able to preserve its successful folding in order to obtain drugs with increasingly better pharmacokinetic and pharmacodynamic profiles.

## Figures and Tables

**Table 1:** Name, sequence and melting temperature (T_m_) of the investigated ODNs. ΔT_m_ indicates the difference between the T_m_ of the modified aptamer and that of TBA. G^Br^, G^OMe^, G^LNA^ and G^F^ indicate 8-bromo-2′-deoxyguanosine, 2’-*O*-methylguanosine, locked nucleic acid guanosine and 2’-F-riboguanosine, respectively.

Name	Sequence	T_m_ (°C) ± 1	ΔT_m_ (°C)
TBA	GGTTGGTGTGGTTGG	50	-
TBABM	G^Br^G^OMe^TTG^Br^G^OMe^TGTG^Br^G^OMe^TTG^Br^G^OMe^	66	+16
TBABL	G^Br^G^LNA^TTG^Br^G^LNA^TGTG^Br^G^LNA^TTG^Br^G^LNA^	71	+21
TBABF	G^Br^G^F^TTG^Br^G^F^TGTG^Br^G^F^TTG^Br^G^F^	89	+39

## Data Availability

Not applicable.

## Supplementary Materials

The following supporting information can be downloaded at: https://www.mdpi.com/article/10.3390/ijms242115529/s1
