# Supplementary material for: Improving the Biological Properties of Thrombin-Binding Aptamer by Incorporation of 8-Bromo-2′-Deoxyguanosine and 2′-Substituted RNA Analogues
Source: Int J Mol Sci. 2023 Oct 24;24(21):15529. doi: 10.3390/ijms242115529 (PMC10647374; doi:10.3390/ijms242115529)
Supplement: Supplementary file 1 [file ijms-24-15529-s001.zip › ijms-2671118-supplementary.pdf]

Supplementary Material

Improving the biological properties of Thrombin Binding Aptamer by 8-bromo-2'-deoxyguanosine and 2'-substituted RNA analogues incorporation.

Antonella Virgilio, Daniela Benigno, Carla Aliberti, Valentina Vellecco, Mariarosaria Bucci, Veronica Esposito* and Aldo Galeone.

Department of Pharmacy, University of Naples Federico II, Napoli, Italy.
* Corresponding author: VE: verespos@unina.it.

Table of contents

PAGE

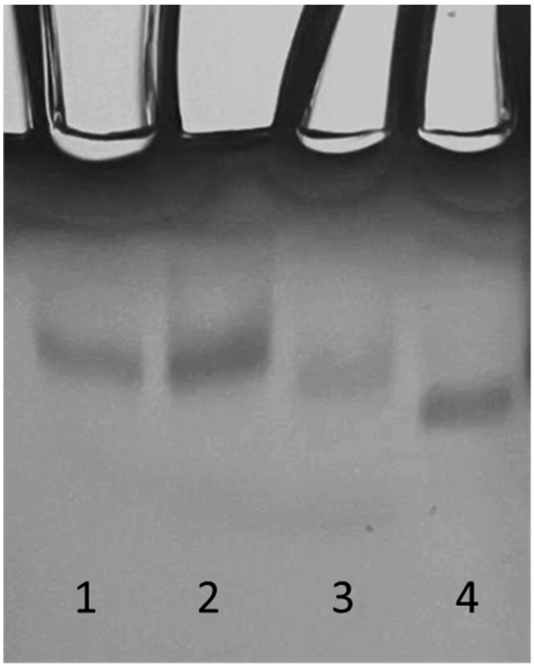

Figure S1. PAGE analysis of TBA and its investigated analogues. Lane 1: TBABM; lane 2: TBABL; lane 3: TBABF; lane 4: TBA. See Materials and Methods for experimental details.
